# Aquaporin-4 IgG antibodies: predictors of positivity and their relationship with neuropsychiatric disorders and white matter lesions in Juvenile systemic lupus erythematosus

**DOI:** 10.1186/s12969-023-00827-6

**Published:** 2023-05-19

**Authors:** Yasmeen Shaaban, Hala EL-Marsafawy, Reham M El-Farahaty, Sherine El-Ziny, Ahmed M EL-Refaey

**Affiliations:** 1 Department of Paediatrics, Mansoura University Children’s Hospital, Mansoura, Egypt; 2 Department of Clinical Pathology, Faculty of Medicine, Mansoura University, Mansoura, Egypt; 3 Faculty of Medicine, Mansoura University, Mansoura, Egypt

**Keywords:** AQP4-Ab, Aquaporin, jSLE, Neuromyelitis optica spectrum disorders (NMOSDs), White matter lesions

## Abstract

**Background:**

This study aimed to describe the prevalence of the various clinical features and severity of juvenile systemic lupus erythematosus (jSLE) and to assess predictors of AQP4-Ab positivity in jSLE. In addition, we assessed the relationship of AQP4-Abs with neuropsychiatric disorders and white matter lesions in jSLE.

**Method:**

For 90 patients with jSLE, demographic data, clinical manifestations, and treatments received were recorded, and all of the patients were underwent clinical examinations, including assessments for the neurological manifestations of jSLE and neuropsychiatric disorders; Systemic Lupus Erythematosus Disease Activity Index (SLEDAI) score evaluations; laboratory investigations, including serum AQP4-Ab assays; and 1.5 Tesla brain MRI. Echocardiography and renal biopsy were performed for the indicated patients.

**Result:**

Fifty-six patients (62.2%) tested positive for AQP4-Abs. These patients were more likely to have higher disease activity scores (p < 0.001); discoid lesions (p = 0.039); neurological disorders (p = 0.001), mainly psychosis and seizures (p = 0.009 and p = 0.032, respectively); renal and cardiac involvement (p = 0.004 and p = 0.013, respectively); lower C3 levels (p = 0.006); white matter hyperintensities (p = 0.008); and white matter atrophy (p = 0.03) than patients who were negative for AQP4-Abs. Furthermore, AQP4-Ab-positive patients were more likely to have received cyclophosphamide (p = 0.028), antiepileptic drugs (p = 0.032) and plasma exchange therapy (p = 0.049).

**Conclusion:**

jSLE patients with higher severity scores, neurological disorders, or white matter lesions could develop antibodies against AQP4. We recommend more studies for systematic screening of AQP4-Ab positivity in jSLE patients to confirm its relationship with neurological disorders.

**Supplementary Information:**

The online version contains supplementary material available at 10.1186/s12969-023-00827-6.

## Introduction

Juvenile-onset systemic lupus erythematosus (jSLE) is a chronic relapsing-remitting systemic autoimmune disease that occurs worldwide and has on onset before 18 years of age. jSLE is a highly complex disease with an unpredictable natural history [1].

jSLE is a multisystem inflammatory disease characterized by an extremely variable presentation and clinical course. Thus, diagnosis can prove challenging. The spectrum of jSLE covers relatively mild disease to severe life-threatening presentations. Major organ inflammation is common. Renal involvement and neuropsychiatric involvement are more frequent in jSLE than in adult-onset disease and can cause significant morbidity [2]. Notable, the incidence of arthritis, nephritis, and neurological involvement in jSLE appears to be negatively correlated with age at disease onset [3] Egyptian children with jSLE appear to have severe disease on presentation, with high SLE Disease Activity Index (SLEDAI) scores, a high prevalence of lupus nephritis (LN) [4], and a high prevalence of neuropsychiatric disorders [5] .

MRI remains the neuroimaging technique of choice due to its superior soft-tissue resolution. MRI can detect brain lesions in neuropsychiatric-SLE (NP-SLE), which shows underlying cerebrovascular and parenchymal brain injury in histopathological analyses [6].

Aquaporin-4 (AQP4) is a water channel widely expressed on the foot processes of astrocytes in the brain and cells in the renal tract, the gastrointestinal system, skeletal muscles, lungs, and blood system. In the central nervous system (CNS), AQP4 is involved in water movement, cell migration, and neuroexcitation [7].

Antibodies against AQP4 (AQP4-Abs) were first identified in 2005 in patients with neuroinflammation who are now best defined as having neuromyelitis optica spectrum disorders (NMOSDs) [8]. These NMOSDs are a group of inflammatory disorders of the CNS that often present with a relapsing-remitting course and are characterized by optic neuritis and longitudinally extensive transverse myelitis [8].

Notably, the presence of AQP4-Abs has already been described in the context of systemic autoimmune diseases such as adult-onset systemic lupus erythematosus (SLE), but their presence in children and adolescents with jSLE is still under investigation. To the best of our knowledge, there is only one known published study discussing this point [9], so the presence of neurotoxic antibodies such as AQP4-Abs remains unclear in jSLE, and further studies are needed.

## Methods

This cross-sectional study was conducted at Mansoura University Children’s Hospital (MUCH) with a convenience sample of 90 jSLE patients recruited from the nephrology outpatient clinic and nephrology unit during the period from December 2019 to December 2020. A total of 26.7% of these patients were newly diagnosed.

Patients diagnosed with jSLE before 18 years of age who met ≥ 4 of the revised (ACR) SLE classification criteria [10] were included in the study. Patients who had other diseases that could explain their ACR SLE classification criteria; patients with spinal lesions and history of recurrent transverse myelitis or optic neuritis; and patients with neuropsychiatric symptoms that could easily be attributed to causes other than jSLE, such as patients with previously existing epilepsy, infection and traumatic brain injury, were excluded.

All patients were subjected to a thorough history taking, and information including age, sex, BMI, therapeutic drugs, and drug adherence was obtained. The clinical examination included a general examination of temperature, blood pressure, and mucocutaneous manifestations (alopecia, malar rash discoid lesions, photosensitivity, ulcerationsand joint examination for signs of arthritis). The cardiovascular system examination included a chest examination. The neurological examination included an evaluation of the patient’s consciousness level, orientation, tone, reflexes and neuropsychiatric manifestations of jSLE (19 syndromes were assessed in our patients, including aseptic meningitis, cerebrovascular disease (CVD), demyelinating syndrome (e.g., multiple sclerosis), headache, movement disorder, myelopathy, seizure disorder, acute confused state, anxiety disorder, cognitive dysfunction, mood disorder, psychosis, Guillain–Barre syndrome, autonomic neuropathy, mononeuropathy, myasthenia gravis, cranial neuropathy, plexopathy, and polyneuropathy) [11]. Disease activity was assessed using the SLEDAI score [12, 13]. Laboratory investigations, including examinations of the complete blood count (CBC), protein values in urine (> 30 mg/dl) were abnormal, erythrocyte sedimentation rate (ESR, first hour; normal < 10 mm/hour), serum C-reactive protein (CRP normal < 10 mg/L), anti-dsDNA (negative < 200 IU/ml), anti-nuclear antibodies (ANA) (negative < 20 IU/ml), C3 (normal range (88–201 mg/dl) and C4 (15–45 mg/dl) levels, lupus anticoagulant (LA) and anticardiolipin (ACL), were performed on all patients. AQP4-IgG antibody assays were performed using a Human Aquaporin-4 Antibody (IgG) ELISA Kit (Bioassay Technology Laboratory, Junjiang International Bldg. 228 Ningguo Rd. Yangpu Dist. Shanghai,China). Renal biopsy and echocardiography were performed for indicated study patients (66 and 52 patients, respectively). A radiological examination (1.5 Tesla) was also performed with the same scanner. The scanning protocol included high-resolution T1-weighted, T2-weighted, and fluid-attenuated inversion recovery (FLAIR) sequences.

### Statistical analysis

Computer-imputed data were analysed using IBM SPSS Statistics for Windows, Version 22.0 (released 2013, IBM SPSS Corp., Armonk, NY). Qualitative data are presented using numbers and percentages. Quantitative data are presented using the median and interquartile range for nonparametric data and the mean and the standard deviation for parametric data after testing for normality using the Kolmogorov‒Smirnov test. Binary stepwise logistic regression analysis was used for the prediction of the independent variables of a binary outcome. Significant predictors in the univariate analysis were entered into the regression model using the forward Wald entry method. Adjusted odds ratios and their 95% confidence intervals were calculated. The significance of the obtained results was judged at the 0.05 level.

## Results

The mean age of the studied patients was 14.46 ± 2.75 years, most of the included patients were females (92.2%), and the mean BMI was 25.23 ± 0.56 kg/m^2^.

The patients were subdivided according to the presence of AQP4-IgG Abs, with 56 (62.2%) patients who were positive and 34 (37.8%) patients who were negative for AQP4-IgG Abs. There was no significant difference between the groups with regard to age, sex, or BMI (Table 1).

**Table 1:** Demographic information, clinical manifestations, SLEDAI scores, and neuropsychiatric disorders among all study patients and subgroups

	All patients (n = 90)	AQP4-IgG Ab negative subgroup (n = 34)	AQP4-IgG Ab positive subgroup (n = 56)	Test of significance
Age/years	14.46 ± 2.75	14.06 ± 2.93	14.69 ± 2.62	p = 0.289
Sex				
Male (%)	7.8	11.8	5.4	P = 0.419
Female (%)	92.2	88.2	94.6	
New patients (%)	26.7	35.3	21.4	p = 0.149
BMI kg/m^2^	25.23 ± 4.33	24.72 ± 4.49	25.54 ± 4.24	p = 0.385
SLEDAI score				
Mild (%)	30.0	52.9	16.1	
Moderate (%)	25.6	32.4	21.4	p < 0.001*
High (%)	32.2	14.7	42.9	
Very high (%)	12.2	0	19.6	
Mood disorders (%)	57.8	50	62.5	p = 0.244
Psychosis (%)	50	32.4	60.7	p = 0.009*
Headache (%)	47.8	35.3	55.4	p = 0.065
Anxiety (%)	47.8	35.3	55.4	p = 0.065
Seizures (%)	31.1	17.6	39.3	p = 0.032*
CVD (%)	26.7	20.6	30.4	p = 0.310
Polyneuropathy (%)	17.8	14.7	19.6	p = 0.553
Acute confusional state (%)	12.2	5.9	16.1	p = 0.152
Movement disorder (%)	10	5.9	12.5	p = 0.310
Cranial neuropathy (%)	10	2.9	14.3	p = 0.082
Cognitive dysfunction (%)	8.9	2.9	12.4	p = 0.122
Aseptic meningitis (%)	7.8	2.9	10.7	p = 0.182
Autonomic neuropathy (%)	5.6	5.9	5.4	P = 1.0
Myelopathy (%)	4.4	2.9	5.4	P = 1.0
Demyelination (%)	1.1	0	2.9	P = 0.378
Myasthenia gravis (%)	1.1	0	2.9	P = 0.378

Data are expressed as numbers (percentages) except age and BMI, for which data are expressed as the mean ± standard deviation. BMI: body mass index. SLEDAI: Systemic Lupus Erythematosus Disease Activity Index

CVD: cerebrovascular disease. p < 0.05 was considered statistically significant

Regarding clinical manifestations, our study revealed that the most common cutaneous manifestations were malar rashes (77.8%), ulcerations (56.7%), alopecia (40%), discoid lesions (20%), and photosensitivity (61.1%), with no significant difference between subgroups except for a significantly higher frequency of discoid lesions in the AQP4-IgG Ab-positive subgroup than in the AQP4-IgG Ab-negative subgroup (p = 0.039). The most common clinical manifestations in the studied patients were renal involvement (73.3%), neurological disorders, arthritis (65.6%), cardiac affection (57.8%), and serositis (51.1%), with a significantly higher frequency of renal involvement, neuropsychiatric disorders and cardiac affection in the AQP4-IgG Ab-positive subgroup than in the AQP4-IgG Ab-negative subgroup (*P* = 0.004, 0.001, 0.04, and 0.013, respectively) (data not shown in table).

Patients were divided on the basis of SLEDAI scores into (30.0%) mild, (25.6%) moderate, (32.2%) high, and (12.2%) very high disease activity subgroups, where the mild disease activity subgroup had SLEDAI scores of 1 to 5, the moderate disease activity subgroup had SLEDAI scores of 6 to 10, the high disease activity subgroup had SLEDAI scores of 11 to 19, and the very disease high activity subgroup had SLEDAI scores ≥ 20 [12]. A comparison of subgroups revealed a significantly higher frequency of high and very high scores in the AQP4-IgG Ab positive subgroup than in the negative subgroup (*P* < 0.004).

There was a significantly higher frequency of seizures and psychosis (*P* = 0.032 and 0.009, respectively) and a trend (approach significant) towards a higher frequency of headache, anxiety, and cranial neuropathy in the AQP4-IgG Ab-positive subgroup than in the AQP4-IgG Ab-negative subgroup (*P =* 0.065, 0.065, and 0.08, respectively). However, there was no statistically significant difference between subgroups regarding other neuropsychiatric manifestations (Table 1).

A statistically significant lower median concentration of C3 and a higher frequency of proteinuria in the AQP4-IgG Ab-positive subgroup than in the AQP4-IgG Ab-negative subgroup were observed (*P* = 0.009 and 0.004, respectively). There were trends towards a higher prevalence of LA and ACL positivity in AQP4-Ab positive patients, but these differences were not statistically significant (*P* = 0.067 and 0.07, respectively). There was no statistically significant difference among subgroups regarding the remaining laboratory investigation parameters, including the WBC count, neutrophil number, lymphocytes number, HB level, platelet count, MPV, ESR, CRP, C4, ANA positivity, and anti dsDNA. The number of patients who needed an echocardiographic examination and the prevalence of pericarditis were significantly higher in the AQP4-Ab-positive subgroup than in the AQP4-Ab-negative subgroup (*P* = 0.013 and 0.018, respectively). There was no statistically significant difference between the two subgroups regarding myocarditis, endocarditis or a valvular effect (Table 2).

**Table 2:** Laboratory and echocardiographic findings among the studied patients and subgroups

	All patients (n = 90)	Anti-AQP4 Ab negative subgroup (n = 34)	Anti-AQP4 Ab positive subgroup (n = 56)	Test of significance
WBC count	6.85 (4.93–9.33)	6.75(2.4–24)	7.0(3.0-26.8)	p = 0.677
Neutrophil number	4.2 (2.8-5)	4.1(1-15.5)	4.2(1.6–18)	p = 0.680
Lymphocyte number	2.0 (1.2–3.5)	1.85(0.4–10)	2.1(0.48–6.3)	p = 0.848
HB level	9.29 ± 1.76	9.52 ± 1.63	9.16 ± 1.84	p = 0.347
Platelet count	190 (117.5-280.25)	189(10–540)	190(63–701)	p = 0.479
MPV	8.7 (7.15–10.83)	8.45(5.4–14.5)	8.75(5-12.6)	p = 0.878
Proteinuria %	73.3	55.9	83.9	p = 0.004*
ESR first hour	104.5 (86-125.25)	65(20–120)	75(35–145)	p = 0.142
CRP	6 (3-18.25)	5.5(1–56)	7.5(1–96)	p = 0.184
C3	53(40.75-63)	60(37.0-137)	48.5(22–108)	p = 0.006*
C4	12.5 (8–17)	12.5(1.0–52.0)	12.5(1.0–45.0)	p = 0.275
ANA %	90 (100%)	34(100)	56(100)	P = 1.0
Anti dsDNA%	70 (77.8%)	27(79.4)	43(76.8)	p = 0.771
LA %	26 (28.9%)	6(17.6)	20(35.7)	p = 0.067
ACL %	63 (70%)	20(58.8)	43(76.8)	p = 0.07
Patients who underwent echocardiography examination %	57.8	41.2	67.9	p = 0.013*
Pericarditis %	42.2	26.5	251.8	p = 0.018*
Valvular %	22.2	17.6	25	p = 0.416
Endocarditis %	11.1	5.9	14.3	p = 0.219
Myocarditis %	8.9	5.9	10.7	p = 0.435

WBC: white blood cell, HB: haemoglobin, MPV: mean platelet volume, ESR: erythrocyte sedimentation rate, CRP: c-reactive protein. C3: complement 3, C4: complement 4, ANA: antinuclear antibody, Anti dsDNA: anti double-stranded antibodies, LA: lupus anticoagulant, ACL: anticardiolipin. p < 0.05 was considered statistically significant

Regarding renal biopsy results, there was no statistically significant difference between the two subgroups regarding disease activity, chronicity index of renal biopsy, and the prevalence of LN classes (Table 3).

**Table 3:** Renal biopsy results among the studied patients and subgroups

	All patients (n = 66)	Anti-AQP4 Ab negative subgroup (n = 19)	Anti-AQP4 Ab positive subgroup (n = 47)	Test of significance
Activity index median (IQR)	5.5(1–8)	2(1–8)	6(2–8)	p = 0.18
Chronicity index median (IQR)	1 (0–2)	1(0-2.5)	1(0–2)	p = 0.892
Class %				
1	7.6	10.5	6.4	
2	10.6	15.8	8.5	MC
3	28.8	26.3	29.8	P = 0.346
4	30.3	15.8	36.2	
5	19.7	31.6	14.9	
6	3.0	0	4.3	

Data are expressed as number (percentage) except those for the activity and chronicity indexes, which are expressed as median (IQR). MC: Monte Carlo test for numbers (percentage) when more than 25% of cells had a count less than 5 in tables (> 2*2). χ^2^ = Chi-square test for numbers (percentage)

**Fig. 1:**
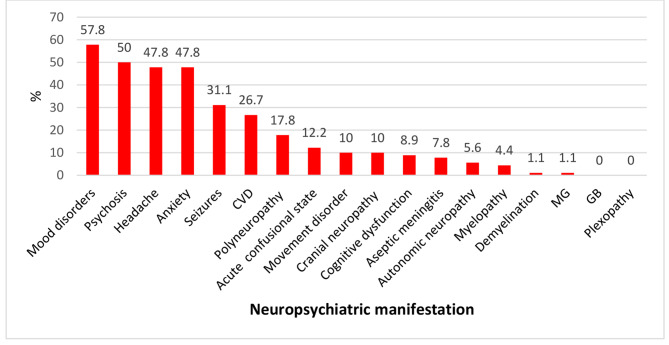
Demonstrates neuropsychiatric manifestation studied in the current JSLE series. CVD: cerebrovascular disease, MG: myasthenia gravis, GB: Guillain-Barré. (CVD: cerebrovascular disease, MG: myasthenia gravis, GB: Guillain‒Barré syndrome)

**Fig. 2:**
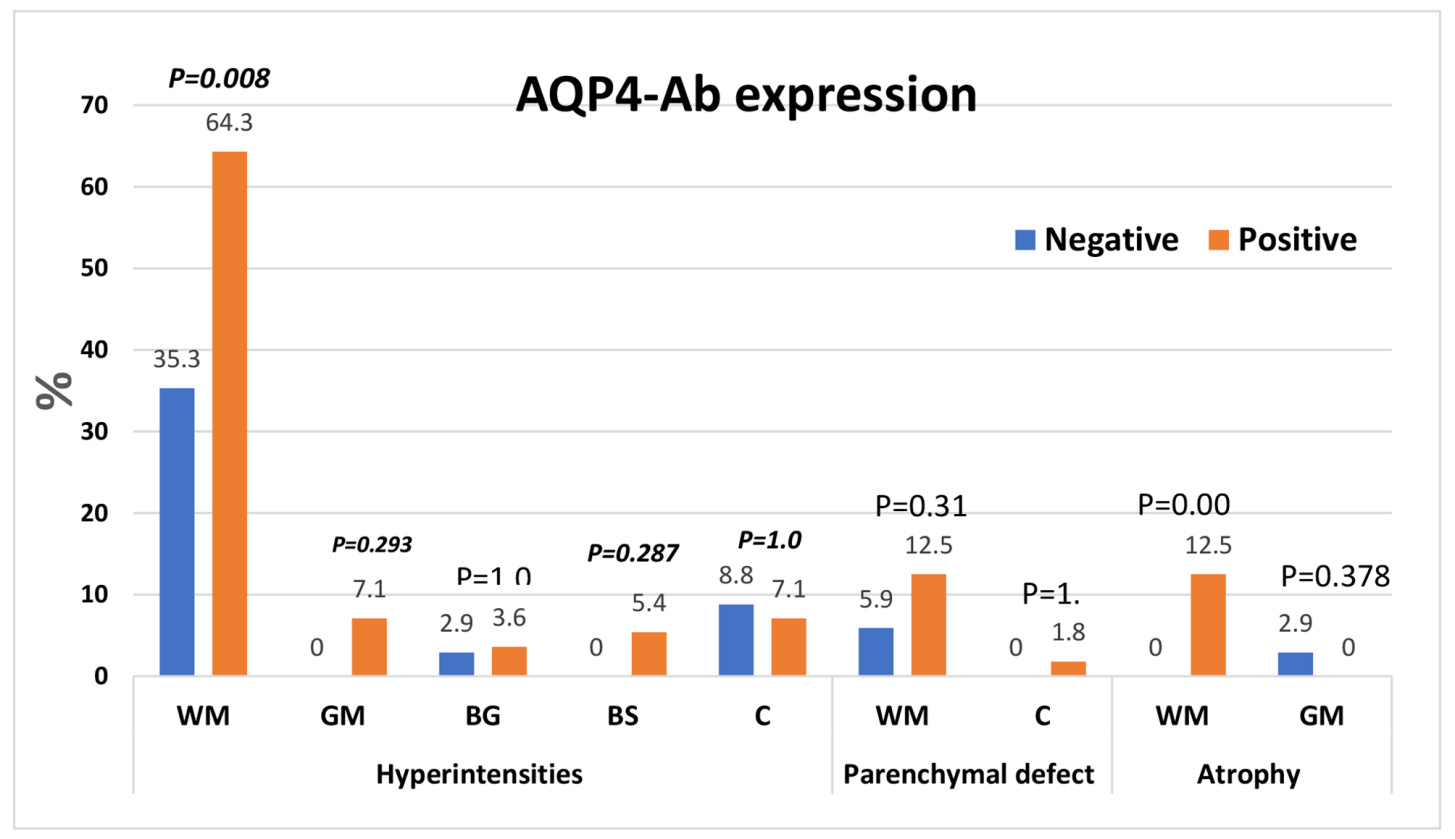
MRI findings in the AQP4-IgG Ab-positive and AQP4-IgG Ab-negative subgroups. WM: white matter, GM: grey matter, BG: basal ganglion, BS: brain stem, C: cerebellum

In this study, the most common brain MRI findings were white matter hyperintensities (53.3%), white matter parenchymal defects (10%), white matter atrophy, and cerebellar hyperintensities (7.8%). The prevalences of white matter hyperintensities and white matter atrophy were significantly higher in the AQP4-Ab-positive subgroup than in the AQP4-Ab-negative subgroup (*P* = 0.008 and 0.032, respectively). However, there was no statistically significant difference between subgroups regarding hyperintensities or parenchymal defects (grey matter, basal ganglia, and cerebellum) (Figs. 1, 2).

Studying the protocols of therapy, we noticed that 86.7% of the patients were receiving maintenance therapy of chloroquine; 70% were receiving cortisone, and 26.7% had been taking cortisone for less than one week; 61.1% were on a cyclophosphamide protocol, and 16.7% had just started their first dose; 34.4% were receiving methotrexate; 24.4% were receiving azathioprine; 32.2% were on an MMF protocol; 57.8% were receiving maintenance antihypertensive medication; 31.1% were receiving maintenance antiepileptic therapy; anticoagulant medications were prescribed to 15.6% of the studied patients and aspirin to 24.4%; and 15.6% had received plasmapheresis. Among the patients, 42.2% did not adhere to their drug therapy, which may be related to a lack of awareness about jSLE complications and the rebellious behaviour of adolescents, which could be aggravated by the mood disorders associated with the disease. There were significantly higher frequencies of maintenance therapy of chloroquine, antiepileptic drugs, cyclophosphamide, azathioprine, plasmapheresis, and drug nonadherence in the AQP4-Ab-positive subgroup than in the AQP4-Ab-negative subgroup (*P =* 0.027, 0.032, 0.028, 0.029, 0.049, and 0.018, respectively). There was a trend towards a higher frequency of cortisone prescription in AQP4-Ab-positive patients, but the difference was not statistically significant (p = 0.06). A statistically insignificant difference was found between the groups regarding methotrexate, MMF, antihypertensive medications, anticoagulant medications, and aspirin prescription.

The prevalences of white matter hyperintensities and white matter atrophy were statistically significantly higher in the AQP4-Ab-positive subgroup than in the AQP4-Ab-negative subgroup (P = 0.008 and 0.032, respectively). However, there was no statistically significant difference between subgroups regarding hyperintensities or parenchymal defects (grey matter, basal ganglia, and cerebellum).

## Discussion

In the current study, the clinical, laboratory, renal biopsy, and echocardiographic examination findings; prescribed medications; MRI findings; and AQP4-IgG Ab status of 90 patients with jSLE were evaluated. To our knowledge, previous studies evaluated AQP4 Ab positivity among adult patients with SLE, either with neuromyelitis optica [9] or in the absence of any CNS symptoms [14], but no studies have evaluated its relationship with disease activity, systemic effects, and white matter hyperintensities in jSLE.

An analysis of the demographic data from the patients in this study revealed that the median age of disease onset was approximately 14 years, with a female predominance, in agreement with data collected worldwide [15–18]. The mean BMI was slightly higher than the normal average for age (25.5 kg/m^2^), especially in the AQP4-IgG Ab-positive subgroup (25.5 kg/m^2^), with no statistically significant difference between the two subgroups, but less than that found in other studies performed in the same region by El Hadidi et al. [5], which could be due to more elderly patients being included in his study. In the current study, mucocutaneous manifestations were the most frequent, which was in agreement with worldwide results reported by Fayez Mohamed et al. [19], Ale’ed and Al-Mayouf [16], and Torrente-Segarra et al. [17].

Mood disorders were the most common neuropsychiatric disorder among patients included in the current study, followed by psychosis, headache, and anxiety. These results were in agreement with those of Torrente-Segarra [17] and Rahman et al. [20] but in contrast to the results of a Canadian study performed by Muscal and Brey [21], who revealed headache as the most common manifestation. The difference between the results of our study and those of Muscal and Brey could be attributable to a lack of psychiatric follow-up in our patients.

An analysis of cardiac involvement in the current patients with SLE revealed that pericarditis was the most frequent (42.2%) finding, followed by valvular affection (22.2%), endocarditis (11.1%) and myocarditis (8.9), which was in agreement with but with lower frequencies than Chang et al. [22], who found pericarditis to be the most common cardiac manifestation among adolescents with jSLE (10.4%), followed by valvular insufficiency (9.1%) Additionally, Torrente-Segarra et al. [17] found that 19.9% of jSLE patients had pericarditis, 4% had valvular insufficiency, 0.6% had myocarditis, and 3.9% had serositis; 51% of the patients included in this study had serositis. This discrepancy could be due to differences in the recruited patients in terms of disease severity and age.

Due to our extensive experience in renal biopsy [23], we conducted the procedure for all eligible patients, resulting in 73.3% of them showing renal involvement. The most common findings in the biopsies were class IV (30.3%) and class III (28.8%) renal involvement.

In this study, 62.2% of the studied patients were positive for AQP4-IgG Abs, and none of them had been diagnosed with NMODS features or transverse myelitis at the time of recruitment to the study. This was in contrast to E Moraitis et al. [9], who found that only 5.5% of their patients tested positive for AQP4-IgG Abs, and all of them had neurological disorders, mainly transverse myelitis and optic neuritis [9]. Another previous study performed by Alexopoulos et al. concluded that AQP4 antibodies could be present in SLE patients and persist for many years, without concurrent clinical or radiological NMOSD signs, and the authors explained this to be due to the induced complement-mediated damage in cultured astrocytes in these patients, similar to the damage induced by antibodies obtained from typical NMO patients [24].

An analysis of the MRI findings of all studied patients revealed that white matter lesions were the most common finding, especially hyperintensities. This finding is similar to that of Al-Obaidi et al., who found white matter hyperintensities on T2-weighted imaging to be the most common lesion (33%), followed by white matter atrophy (18.5%) and grey matter lesions (3%) [25]. Additionally, Demirkaya et al. found small white matter lesions consistent with acute ischaemia as the most common finding in brain MRI of jSLE patients [26]. The frequencies of white matter hyperintensities and white matter atrophy were significantly higher in the AQP4-Ab-positive subgroup, which could be explained by the higher disease activity and presence of neuropsychiatric symptoms in the AQP4-Ab-positive subgroup, which is probably associated with a significantly higher frequency of white matter changes, as previously observed by Zaky et al. [27]. Moreover, it is well known that AQP4,as one of the water channel proteins on the plasma membrane of astrocytes, is upregulated in various conditions associated with brain oedema in various inflammatory lesions [28]. AQP4 is also present in astrocyte endfeet and plays a role in astrocyte migration and hypertrophy. This relationship between neuroinflammation and AQP4 was explained in research on neuromyelitis optica (NMO), in which serum antibodies were found to recognize astrocytic AQP4 and to be associated with oedema in human autopsied brains; in this research, it was found that AQP4 upregulation was consistently found in astrocytes in various inflammatory lesions [28, 29]. A previous study of MRI findings in the CNS and aquaporin-4 autoimmunity showed that 29% of the patients presented with extensive hemispheric effects that were related to high Ab titres and cerebral deep white matter (58%) [30].

Regarding drug therapy, a large percentage of the patients were not adherent to their prescribed drug regimens, which was also reported by Davis et al. [31]. In this study, a statistically significant difference between subgroups was found regarding antiepileptic drug and cyclophosphamide prescription as well as the frequency of patients who had received plasma exchange therapy, all of which were higher in the AQP4 Ab-positive subgroup. No statistically significant difference was found between subgroups regarding patients receiving hydroxychloroquine, cortisone, antihypertensive medication, methotrexate, azathioprine, mycophenolate mofetil, aspirin, or anticoagulant drugs. In contrast, E Moraitis et al. [9] found a statistically significant difference between subgroups regarding hydroxychloroquine and anticoagulant therapy plus antiepileptic therapy; this difference may be due to a higher number of patients in the AQP4 Ab-negative group in the current study.

## In conclusion

We found an association between AQP4 Ab positivity and lupus neuropsychiatric disorders as well as white matter lesions. Additionally, there was an association between disease activity and these autoantibodies. Therefore, we recommend further studies to validate our observations and to clarify whether we can use AQP4 Ab positivity as a reliable marker for SLE activity and the possibility of neurological effects.

## Electronic supplementary material

Below is the link to the electronic supplementary material.

Supplementary Material 1

Supplementary Material 2

## Data Availability

Data is available upon request.

## Abbreviations

jSLE: juvenile systemic lupus erythematosus
SLEDAI: Systemic Lupus Erythematosus Disease Activity Index
AQP4-Ab: Aquaporin-4 Antibodies
NMOSDs: neuromyelitis optica spectrum disorders
LN: lupus nephritis
NP-SLE: neuropsychiatric-SLE
MRI: Magnetic resonance imaging
CNS: central nervous system
MUCH: Mansoura University Children’s Hospital
ACR: American College of Rheumatology
CVD: cerebrovascular disease
CBC: complete blood count
ESR: erythrocyte sedimentation rate
CRP: serum C-reactive protein
ANA: anti-nuclear antibodies
LA: lupus anticoagulant
ACL: anticardiolipin
ELISA: enzyme-linked immunosorbent assay
FLAIR: fluid-attenuated inversion recovery
BMI: body mass index
WBC: white blood cell
HB: haemoglobin
MPV: mean platelet volume
C3: complement 3
C4: complement 4
MMF: Mycophenolate mofetil
