# Supplementary material for: Aquaporin-4 IgG antibodies: predictors of positivity and their relationship with neuropsychiatric disorders and white matter lesions in Juvenile systemic lupus erythematosus
Source: Pediatr Rheumatol Online J. 2023 May 19;21:47. doi: 10.1186/s12969-023-00827-6 (PMC10197312; doi:10.1186/s12969-023-00827-6)
Supplement: Supplementary file 1 — Supplementary Material 1 [file 12969_2023_827_MOESM1_ESM.pdf]

SPRINGER NATURE
Author Services

Editing Certificate

This document certifies that the manuscript

Aquaporin-4 IgG Antibody: Predictors of Positivity, Relation to Neuropsychiatric Disorders and White Matter Lesions in Juvenile Systemic Lupus Erythematosus.

prepared by the authors

Yasmeen Shaaban MD1, Ahmed M EL-Refaey PhD1, Hala EL-Marsafawy PhD1, Reham M El-Farahaty PhD2, Sherine El-Ziny1 Nephrology Unit, Mansoura University Children's Hospital, Mansoura, EGYPT Department of Pediatrics 1, Department of Clinical Pathology 2, Faculty of Medicine - Mansoura University, Mansoura, EGYPT

was edited for proper English language, grammar, punctuation, spelling, and overall style by one or more of the highly qualified native English speaking editors at SNAS.

This certificate was issued on **September 6, 2022** and may be verified on the SNAS website using the verification code **F42E-D0CC-8703-6218-C2A9**.

Neither the research content nor the authors' intentions were altered in any way during the editing process. Documents receiving this certification should be English-ready for publication; however, the author has the ability to accept or reject our suggestions and changes. To verify the final SNAS edited version, please visit our verification page at secure.authorservices.springernature.com/certificate/verify.
If you have any questions or concerns about this edited document, please contact SNAS at support@as.springernature.com.

SNAS provides a range of editing, translation, and manuscript services for researchers and publishers around the world.
For more information about our company, services, and partner discounts, please visit authorservices.springernature.com.
