# Supplementary material for: Aquaporin-4 IgG antibodies: predictors of positivity and their relationship with neuropsychiatric disorders and white matter lesions in Juvenile systemic lupus erythematosus
Source: Pediatr Rheumatol Online J. 2023 May 19;21:47. doi: 10.1186/s12969-023-00827-6 (PMC10197312; doi:10.1186/s12969-023-00827-6)
Supplement: Supplementary file 2 — Supplementary Material 2 [file 12969_2023_827_MOESM2_ESM.docx]

**Cover letter**

Authors:

**Yasmeen Shaaban, Ahmed M El-Refaey, Hala EL-Marsafawy, Reham M El-Farahaty, Sherine El-Ziny**

**Corresponding author:**

**Ahmed M El-Refaey.**

**E-mail: arefaey72@mans.edu.eg**

**Prof Alberto Martini, University of Genoa, Italy. (Editor-in-Chief: Pediatric rheumatology journal).**

**Prof Charles Spencer, Ohio State University, USA (Editor-in-Chief: Pediatric rheumatology journal).**

Dear professors:

I would be grateful if you could consider our article titled:

**Aquaporin-4 IgG Antibodies: Predictors of Positivity and Their Relationship with Neuropsychiatric Disorders and White Matter Lesions in Juvenile Systemic Lupus Erythematosus.** For publication in the ***Pediatric rheumatology journal***

Best wishes.

Sincerely,

Corresponding author:

**Ahmed Mahmoud EL-Refaey PhD (EL-Refaey AM)**

**Professor of Paediatrics, Nephrology Unit, Department of Paediatrics,** **Mansoura University Children's Hospital, Mansoura. EGYPT**

**Address of the corresponding author:**

**Nephrology Unit, Department of Paediatrics, Mansoura University Children's Hospital, Mansoura. EGYPT**

**Mobile phone:01001989292**

**E-mail: arefaey72@mans.edu.eg**
